# Gestational trophoblastic neoplasia with retroperitoneal metastases: A fatal complication

**DOI:** 10.1186/1477-7819-8-114

**Published:** 2010-12-30

**Authors:** Nikolaos Thomakos, Alexandros Rodolakis, Panayiotis Belitsos, Flora Zagouri, Ioannis Chatzinikolaou, Athanassios-Meletios Dimopoulos, Christos A Papadimitriou, Aris Antsaklis

**Affiliations:** 1 1st Department of Obstetrics and Gynaecology, University of Athens, Alexandra Hospital, Greece; 2 Department of Clinical and Therapeutics, Alexandra Hospital, School of Medicine, University of Athens, Greece

## Abstract

**Background:**

Gestational Trophoblastic Neoplasia (GTN) is a pathologic entity that can affect any pregnancy and develop long after the termination of the pregnancy. Its course can be complicated by metastases to distant sites such as the lung, brain, liver, kidney and vagina. The therapeutic approach of this condition includes both surgical intervention and chemotherapy. The prognosis depends on many prognostic factors that determine the stage of the disease.

**Case Report:**

We present a woman with GTN and retroperitoneal metastatic disease who came to our department and was diagnosed as having high risk metastatic GTN. Accordingly she received chemotherapy as primary treatment but unfortunately developed massive bleeding after the first course of chemotherapy, was operated in an attempt to control bleeding but finally succumbed.

**Conclusion:**

This case demonstrates that GTN, while usually curable, can be a deadly disease requiring improved diagnostic, treatment modalities and chemotherapeutic agents. The gynaecologist should be aware of all possible metastatic sites of GTN and the patient immediately referred to a specialist center for further assessment and treatment.

## Introduction

Gestational Trophoblastic Neoplasia (GTN) refers to a pathologic condition that is characterized by aggressive invasion of the endometrium and myometrium by trophoblastic cells and is divided to four different pathologic entities: *invasive mole*, *gestational choriocarcinoma*, *placental site trophoblastic tumour *and *epithelioid trophoblastic tumour *[1]. GTN typically develops with or follows some form of pregnancy, but occasionally an antecedent gestation cannot be confirmed with certainty. Most cases follow a hydatidiform mole. Rarely, GTN develops after a live birth, miscarriage, or termination [2].

Metastases in GTN develop in about 4% after evacuation of a complete mole [3] but are more often seen when GTN develops after non-molar pregnancies. High propensity of distant metastases characterizes gestational choriocarcinoma which develops in approximately 1 in 30,000 non-molar pregnancies [3-5]. Two thirds of such cases follow term pregnancies, and a third develops after a spontaneous abortion or pregnancy termination [3]. Choriocarcinoma should be suspected in any woman of reproductive age with metastatic disease from an unknown primary [4,5]. Moreover, it may be suspected in any abnormal bleeding for more than 6 weeks following a pregnancy; in this case human Chorionic Gonadotrophin (hCG) testing is indicated to exclude a new pregnancy or GTN [4,5]. Gestational choriocarcinomas initially invade the endometrium and myometrium although blood-borne systemic metastases tend to develop early during the course of the disease [6]. This can be explained by the great vascularity of trophoblastic tumours which causes them to bleed profusely [6]. The diagnosis of choriocarcinoma is often delayed due to subtle signs and symptoms of disease in patients with an antecedent normal pregnancy. Therefore choriocarcinoma has a significantly higher mortality rate than GTN following non-molar abortions; this rate reaches up to 14% [4,7,8].

We present a rare case of GTN with retroperitoneal metastases and uncontrollable bleeding after the first cycle of chemotherapy.

## Case Report

A G3P1 39-year-old woman of Asian origin complaining of diffuse abdominal pain and vaginal bleeding was referred to our department with ultrasound findings compatible with molar pregnancy. Obstetrical history included Caesarean Section 4 years ago and a termination of pregnancy within the last six months. Her last menstrual period was 14 weeks before admission.

**Figure 1:**
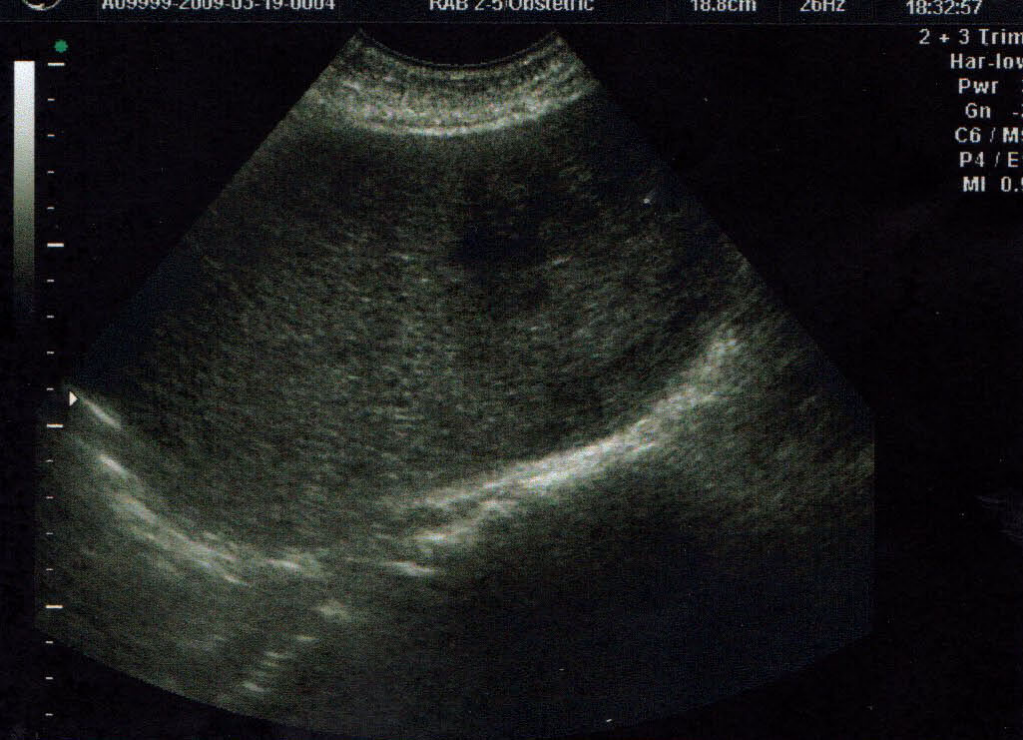
**Ultrasound showing uterus content compatible with molar pregnancy**.

**Figure 2:**
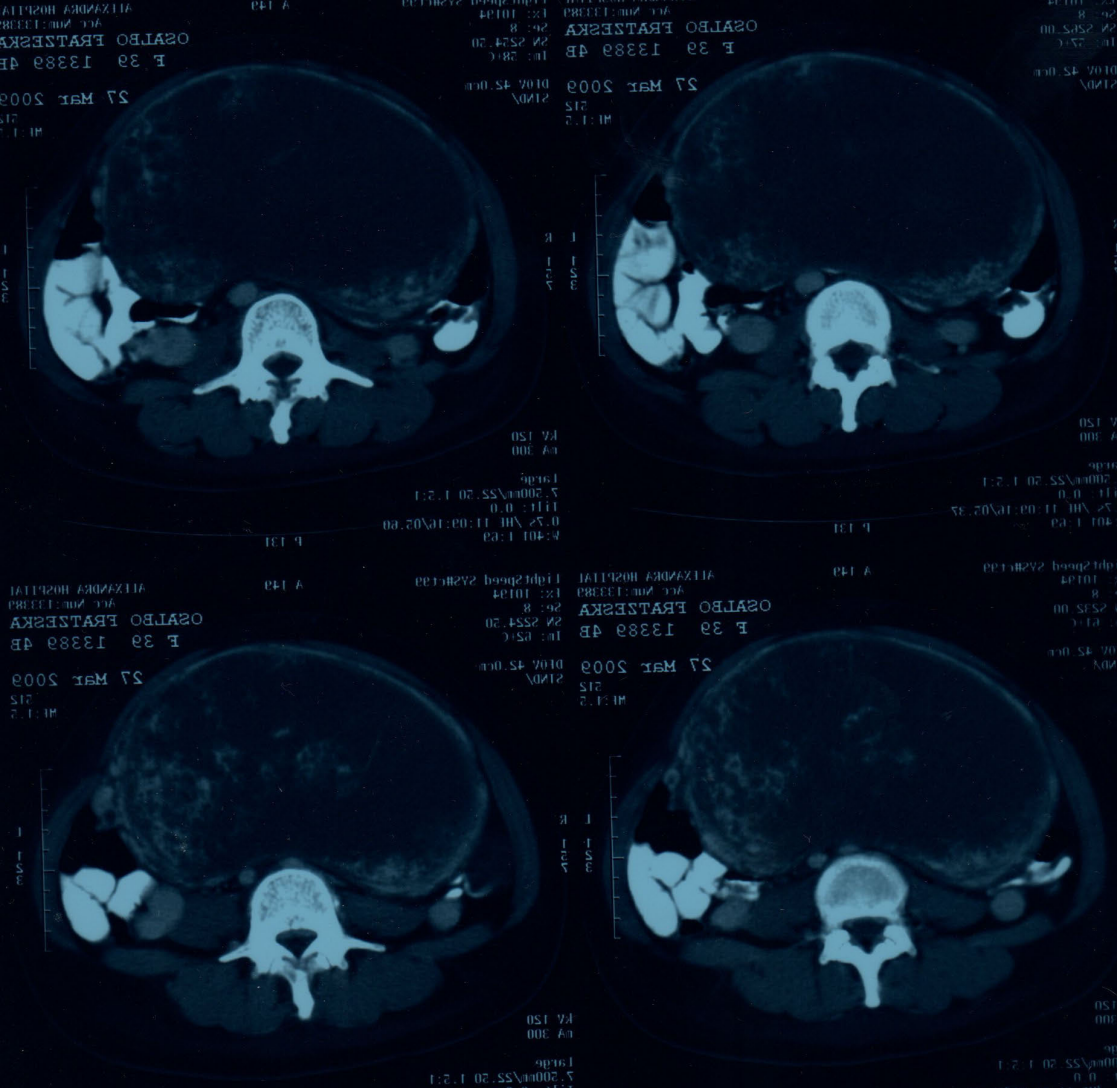
**Abdominal CT showing occupation of the uterus by the trophoblastic tissue**.

**Figure 3:**
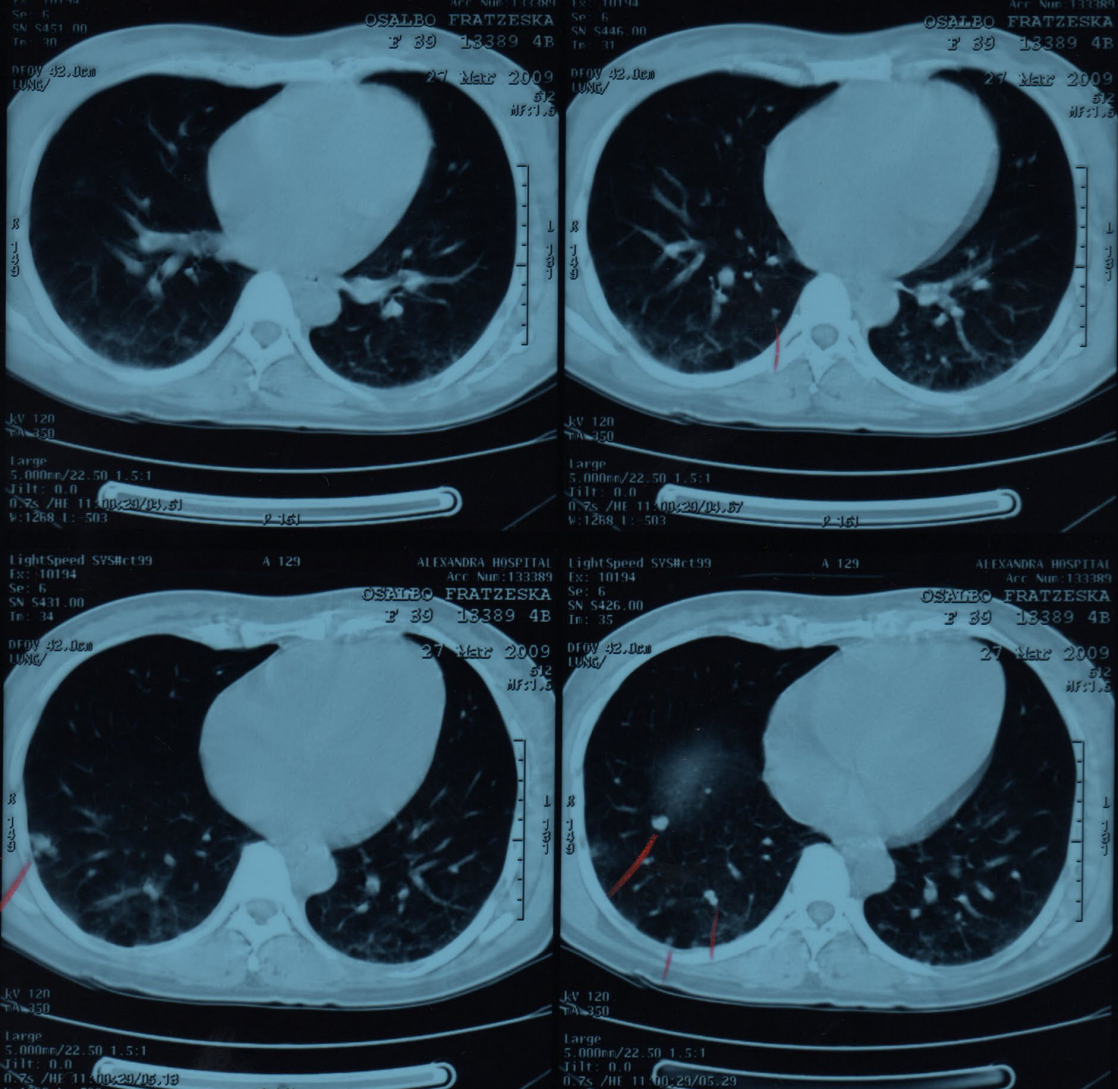
**Chest CT showing the multiple metastases of the trophoblastic disease**.

Physical examination revealed uterine size that corresponded to 18 weeks pregnancy and the content was compatible with molar pregnancy (Figure 1). The laboratory workup revealed profound anaemia (Haematocrit 26%) and β-hHG >22.500. The Computerised Tomography of the abdomen confirmed the findings of the ultrasound scan while the chest CT showed multiple nodular lesions in both lung fields (Figure 2, 3). The patient was classified as high risk (total score 9) according to the WHO Prognostic Scoring System for Gestational Trophoblastic Disease (GTD).

She initially underwent dilatation and curettage which was incomplete and had to be repeated after 2 days. Histology showed complete molar pregnancy. According to FIGO staging system the patient was stage III high-risk invasive GTD; therefore chemotherapy was scheduled. The chemotherapy regimen included Bleomycin, Etoposide and CDDP given for 4 cycles. Each cycle included 3 days Bleomycin 15 mg/day, 3 days of Etoposide 120 mg/m2 and 2 days of CDDP 50 mg/m2.

One day after the first cycle of chemotherapy she started to complain of diffuse abdominal pain and within 10-15 minutes she lost consciousness and had a cardiac arrest but she was successfully resuscitated and because of the continuous drop in the Haematocrit and abdominal paracentesis positive for blood, it was decided to proceed with explorative laparotomy.

During laparotomy diffuse retroperitoneal bleeding was encountered from multiple microscopic amd macroscopic sites. A general surgeon was called to assist the operating team. Initially the gynecological team tried to put some sutures for hemostasis in macroscopically bleeding sites, especially in the subdiaphragmaic areas. In an attempt to control bleeding splenectomy was performed. Hemorrhage control was impossible and the retroperitoneal space was tamponaded in order to stabilize the patient and re-operate her after 48 hours. The patient was then transferred to the ICU where she continued to bleed. She received a total of 26 concentrated red blood cell units as well as fresh frozen plasma, concentrated coagulation factors, colloids and even somatostatin in order to stabilize or reverse her deteriorating condition. Unfortunately she finally developed DIC due to uncontrollable bleeding and died after 3 days.

## Discussion

Choriocarcinomas originating from complete molar gestations comprise most cases of metastatic GTN [2]. Four to five percent of complete moles develop metastatic choriocarcinoma after evacuation and chemotherapy is indicated whenever choriocarcinoma is diagnosed histological [2]. Although many patients are largely asymptomatic, metastatic GTN is highly vascular and prone to severe haemorrhage either spontaneously or during biopsy. The most common sites of metastases are the lungs (80%), vagina (30%), pelvis (20%), liver (10%), and brain (10%) [9].

As concerns the clinical manifestations, menorrhagia is the most common presenting symptom. Patients with pulmonary metastases typically have asymptomatic lesions identified on routine chest radiograph and infrequently present with cough, dyspnoea, haemoptysis, or signs of pulmonary hypertension [10]. Hepatic or cerebral involvement is encountered almost exclusively in patients who have had an antecedent non-molar pregnancy and a protracted delay in tumour diagnosis [11]. These women may present with associated haemorrhagic events. Virtually all patients with hepatic or cerebral metastases have concurrent pulmonary or vaginal involvement or both [9]. Great caution is used in attempting excision of any metastatic disease site due to the risk of profuse haemorrhage [2,9,12]; therefore metastasis should generally not be resected. Thus, this practice is almost uniformly avoided except in extenuating circumstances of life-threatening brain stem herniation or chemotherapy-resistant disease.

Patients with GTN should undergo a thorough pretreatment assessment to determine the extent of disease. The initial evaluation includes a pelvic examination, chest radiograph, and ultrasound or abdominal-pelvic CT scan [13,14]. Although the chest radiographs are considered adequate for lung metastases detection, frequently lesions can be missed on conventional radiographs and a CT scan is recommended to confirm pulmonary metastatic disease. In addition, positron-emission tomography/computed tomography (PET/CT) may be useful in the evaluation of occult choriocarcinoma when conventional imaging fails to identify metastatic disease [15]. Other diagnostic workups should include liver function tests.

World Health Organization (WHO) modified the prognostic scoring system in an attempt to distinguish patients at low risk for therapeutic failure from those at high risk. Women with high risk scores are more likely to have tumors that are resistant to single-agent chemotherapy. They are therefore treated initially with combination chemotherapy.

Treatment is initiated primarily with chemotherapeutic agents [9]. Gordon et al found that patients with score of at least 8 required multiagent chemotherapy [16]. Bagshawe et al in 1989 found that medium and high risk patients (score 5 or more) should also receive multiagent chemotherapy [17]. Chemotherapy for High-Risk GTN includes Etoposide, methotrexate, and dactinomycin alternating with cyclophosphamide and vincristine (EMA/CO) which is a well-tolerated and highly effective (83% survival rate and cure rate as high as 100%) regimen for high-risk GTN that should be considered the primary treatment in most circumstances. The regimen is repeated every 14 days [9].

EMA-CO remains the preferred chemotherapy for management [18-22], however other regimens like EMA-EP (etoposide, ethotrexate, actinomycin and cisplatinum), PVB (cisplatin, vinblastine and bleomycin), and BEP (bleomycin, etoposide and cisplatin) are widely used especially as second-line therapy in women who experienced resistance to primary chemotherapy [18-22]. Moreover, vincristine/actinomycin D/cyclophosphamide (VAC) or vincristine/iphosphamide/cisplatin (VIP) have been reported to be used as third line treatment [19,22]. In our patient we preferred to use BEP regimen; a well tolerated combination with approved efficacy [18-22]. Optimization of treatment strategies for those who develop drug resistance remains a key challenge; therefore different regimens and multiple combinations are widely used [18,19,22].

Response rates are comparable whether patients are treated primarily or after failure of single-agent chemotherapy and complete remission rates vary between 70% and 80% [23-25]. Repeat dilatation and curettage is generally avoided to prevent morbidity and mortality caused by uterine perforation, haemorrhage, infection, uterine adhesions, and anaesthetic complications [5]. The chemotherapy agents are administered until three negative test results for β-hCG are achieved for three consecutive weeks or until serious side effects develop [12]. After the return to normal β-hCG levels two more courses of chemotherapy can be given as consolidation therapy [12]. Hysterectomy may play a role in the treatment of GTN. First, it may be performed primarily to treat placental site trophoblastic tumours, epithelioid trophoblastic tumours, or chemotherapy-resistant disease. In addition, severe uncontrollable vaginal or intra-abdominal bleeding may necessitate hysterectomy as an emergency procedure [26].

Pulmonary metastases in most cases are treatable with combined chemotherapy but sometimes it is necessary to perform thoracotomy in order to remove a pulmonary module that does not resolve after chemotherapy [12]. Brain metastases are treated by radiotherapy along with chemotherapy. Chemotherapy alone can also be used for brain disease [27]. The primary target of radiation is to reduce the incidence of spontaneous intracranial bleeding during or more commonly after chemotherapy [12,28]. Occasionally, craniotomy has to be performed in order to decompress the brain after an acute cerebral haemorrhage [28]. Liver metastasis is managed with systemic chemotherapy but intra-arterial chemoinfusion has been tried as well [9]. Liver metastasis radiation can also be applied [29]. It may sometimes be necessary to resect a part of the liver in order to remove a persistent metastasis or to control a haemorrhagic area [9].

Another treatment option is to start chemotherapy in GTN with massive disease or metastatic sites likely to bleed profusely (such as liver and brain) with a single or double agent rather than multi-agent treatment [1,2,18,19]. This can allow stabilization of the patient and gradual response to the chemotherapy avoiding rapid tumour necrosis which could lead to respiratory function deterioration and excessive bleeding from metastatic sites [1,2,18,19].

The presence of retroperitoneal diffuse metastases has seldom been described and the clinician must be aware of this extremely rare but fatal complication of metastatic GTD since its course is initially insidious and can rapidly lead to massive haemorrhage and death. The presence of such metastases in our patient caused massive bleeding after the completion of the first cycle of chemotherapy rendering her haemodynamically unstable. The fact that these metastases were diffuse made it impossible for us to successfully control bleeding during laparotomy and this had as a consequence the disseminated intravascular coagulation of the patient.

In conclusion it seems that this death was probably was a failure of the diagnosis of retroperitoneal disease. It therefore becomes obvious that better diagnostic, treatment modalities and chemotherapeutic agents will help to improve control of the disease and increase patient survival. The gynaecologist should be aware of all possible metastatic sites of GTN, moreover he should be alert and capable of identifying this fatal complication as soon as possible. In this case the patient should immediately be referred to a specialist center for further assessment and treatment.

## Competing interests

The authors declare that they have no competing interests.

## Authors' contributions

NT: assisted in the writing of the manuscript and in the gynecological work-up of the patient; PB: assisted in the drafting of the manuscript and made PubMed research; AR: performed the gynecological work-up of the patient and revised critically the manuscript; FZ: made PubMed research and assisted in the writing of the manuscript; IC: assisted in the chemotherapy administration; AMD: decided for the chemotherapy regimens administration and evaluated critically the manuscript; CP: decided for the chemotherapy regimens administration and evaluated critically the manuscript; AA: evaluated critically the manuscript and gave final approval for the manuscript to be published. All authors read and approved the final manuscript.

## Informed consent

Written informed consent was obtained from the patient for publication of this case report and accompanying images. A copy of the written consent is available for review by the Editor-in-Chief of this journal.
